# Risk prediction for cardiovascular related diseases using PRS and EHR in the Framingham Heart Study

**DOI:** 10.1371/journal.pone.0345914

**Published:** 2026-04-17

**Authors:** Taegun Kim, Jaeseung Song, Jong Wha J. Joo

**Affiliations:** 1 Department of Computer Science and Engineering, Dongguk University-Seoul, Seoul, South Korea; 2 Department of Life Sciences, Dongguk University, Seoul, South Korea; 3 Department of Computer Science and Artificial Intelligence, Dongguk University, Seoul, South Korea; Universita degli Studi di Roma Tor Vergata, ITALY

## Abstract

Cardiovascular disease is a leading cause of mortality and rising healthcare costs worldwide. Fortunately, the disease is preventable, and addressing risk factors can significantly reduce its effects. Over the past decade, risk prediction models have advanced significantly, with polygenic risk scoring analysis, which is often used in combination with clinical health information for prediction. However, most previous cardiovascular disease prediction studies based on polygenic risk scores have focused on a single specific disease or event, such as cardiac events. Given the complex nature of the cardiovascular disease, which involves a combination of genetic and environmental factors, a comprehensive analysis of the disease prediction results is essential. In this study, we investigate the genetic and environmental factors contributing to cardiovascular disease by utilizing data from the Framingham Heart Study, a leading cardiovascular cohort. We compared the prediction performance of different methods across various scenarios and assessed performance using various evaluation metrics to identify the best-fitting model for six cardiovascular related diseases. We also analyzed the feature importance of genetic and clinical variables, noting that different variables had varying effects on each disease. Our findings demonstrated the performance of prediction algorithms in forecasting cardiovascular disease by utilizing genetic and clinical factors, as well as highlighting the importance of each feature in the disease prediction. While models relying solely on polygenic risk score showed relatively low prediction performance for some diseases, integrating genetic information with clinical data improved prediction performance in most cases. For certain diseases, particularly those known to be heritable, polygenic risk scores demonstrated predictive ability, suggesting that they may serve as standalone predictive tools. We believe our study reveals the value of combining polygenic risk scores with clinical variables and expect that our thorough analysis can inform study designs tailored to specific diseases and research objectives.

## Introduction

Cardiovascular disease (CVD) is one of the most prevalent diseases in modern society, posing a significant health risk and a substantial financial burden on global healthcare systems [1–13]. CVD is a complex disorder resulting from interactions between genetic and environmental factors; however, the specific contributions of genes and environmental influences remain poorly understood. CVD encompasses a variety of conditions affecting the heart and blood vessels, including myocardial infarction, coronary heart disease (CHD), arrhythmia, and heart failure. Moreover, CVD has a substantial influence on major adult diseases including diabetes and dementia that can precede or follow it [14–20]. Although CVD is largely preventable, many individuals remain unaware of their risk because early stages may be asymptomatic or present with nonspecific symptoms that overlap with other diseases.

Over the past decade, risk prediction models have advanced considerably. Early-stage CVD risk prediction models typically rely on linear multivariate regression methods, which generally exhibit only moderate prediction performance for specific subpopulations [1,21,22]. However, with the rise of machine learning (ML) technologies, newer models have enhanced CVD risk predictions by analyzing larger datasets and identifying the complex interactions among predictors [21]. These approaches have employed various algorithms, including random forest, gradient boosting machine, Naive Bayes, support vector machines, and artificial neural networks, often in combination as ensembles to enhance prediction accuracy [23–28].

Genome-wide association studies (GWAS) have identified numerous genetic variants associated with CVD. The polygenic nature of complex diseases, such as CVD, involves multiple genetic variants, each contributing a small effect that collectively influences trait variability [29]. Polygenic risk scores (PRS) quantify an individual’s genetic risk by aggregating the effects of multiple variants, thereby improving disease prediction. Recognizing the substantial role of environmental factors in CVD susceptibility, studies aim to enhance PRS performance by incorporating data from multi-traits [30,31], disease-related biomarkers [32,33], clinical risk factors [34–36], and environmental variables [32–35] that influence disease risk. However, most previous CVD prediction studies based on PRS focus on one specific disease or event, such as cerebrovascular [37], coronary artery disease [38–42], diabetes [43,44], or cardiac events [45], without thoroughly investigating the genetic and environmental factors associated with overall CVD-related diseases. Given the complex nature of CVDs, which result from a combination of genetic and environmental factors, a detailed analysis of CVD prediction outcomes is essential.

This study aimed to investigate multiple CVD-related phenotypes and assess the relative importance of predictive variables, comparing the contribution of genetic and clinical measurements for each phenotype. Six cardiovascular-related diseases atrial fibrillation (AF), stroke, CHD, congestive heart failure (CHF), dementia, and diabetes were analyzed using data from the Framingham Heart Study (FHS), a long-term, large-scale representative cardiovascular cohort. PRS was calculated to estimate genetic risk using FHS genotypes and GWAS summary statistics from the GWAS catalog. Electronic health records (EHR) from the FHS provided clinical variables for risk prediction. To evaluate the contributions of genetic and environmental factors, prediction performance was assessed under three scenarios: PRS-only, EHR-derived clinical variables-only, and an integrated model combining PRS and EHR variables. Logistic regression and four representative ML methods random forest, XGBoost [46], CatBoost [47], and LightGBM [48] were employed to predict each disease and identify the most suitable model. The Shapley Additive Explanations (SHAP) framework was used to quantify the influence of genetic and clinical variables on the prediction of each phenotype.

## Materials and methods

### Overview of the study design

**Fig 1:**
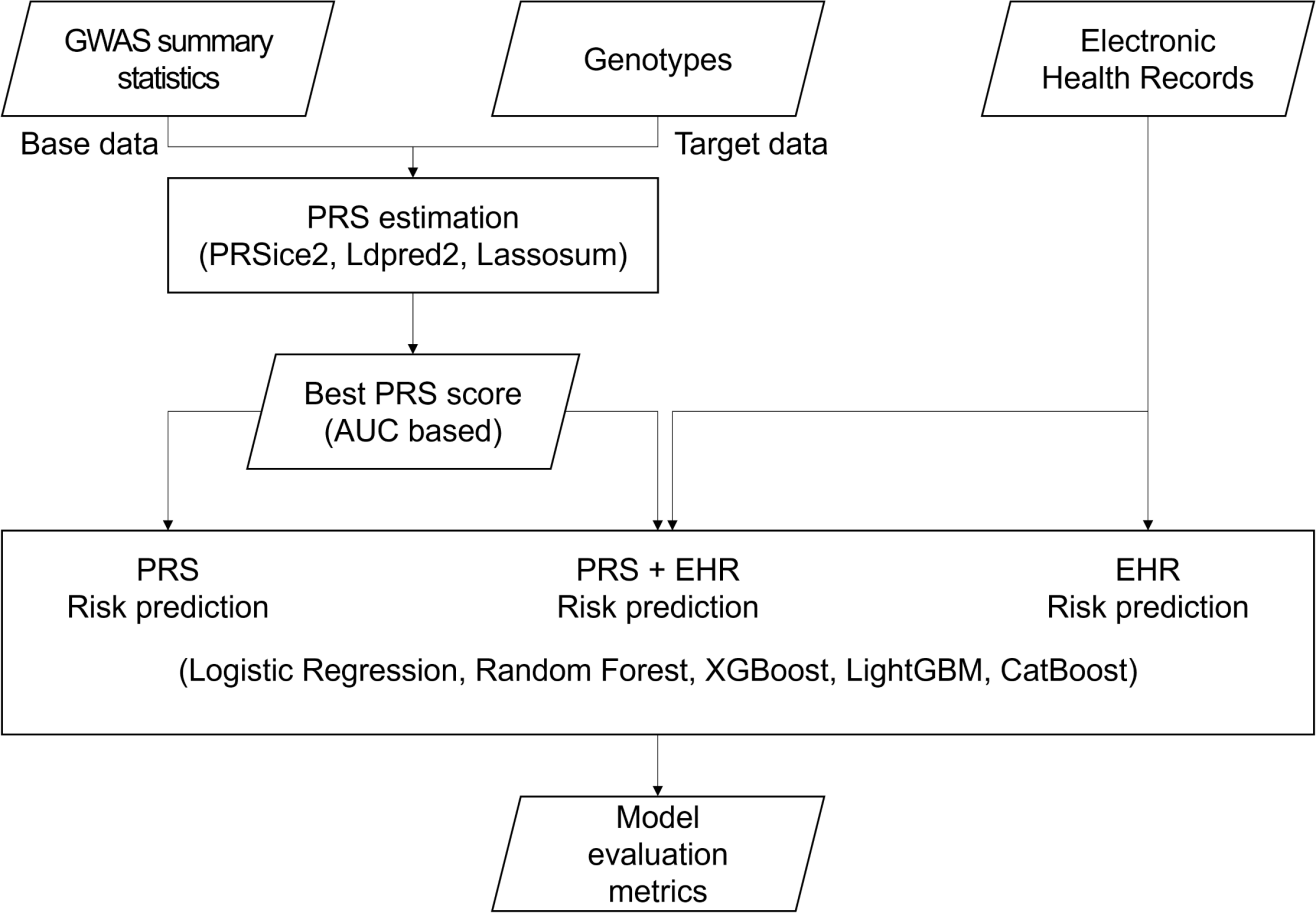
Workflow of risk prediction analysis. GWAS summary statistics from the GWAS catalog and genotypes from the FHS are used as base and target data, respectively, for PRS estimation. PRS scores were calculated using PRSice2, LDpred2, and Lassosum, with prediction accuracy assessed by area under the receiver operating characteristic curve (AUC) in logistic regression. The best-performing PRS method is selected for each disease and used for risk prediction with logistic regression and four ML methods, under three scenarios: PRS-only, PRS + EHR, and EHR. Model evaluation metrics are calculated for each scenario and disease outcome.

The study aims to systematically evaluate the predictive utility of PRS and EHR for CVD-related disease risk prediction by comparing different models. PRS was calculated for six disease outcomes AF, stroke, CHD, CHF, dementia, and diabetes using three widely adopted PRS methods: PRSice2 [49], LDpred2 [50], and Lassosum [51]. For each disease, PRS predictive performance was initially compared using logistic regression and the method with the highest accuracy was selected as the disease-specific genetic risk score model. Using the selected PRS, three prediction scenarios were evaluated: (1) PRS-only (2) EHR-only (3) PRS + EHR. This framework enabled a comprehensive assessment of the standalone predictive value of PRS and its contribution beyond conventional clinical risk factors. Across these scenarios, logistic regression and four ML methods random forest, XGBoost, LightGBM, and CatBoost were applied for risk prediction. Fig 1 illustrates the overall streamline of our analysis.

### CVD data from FHS and GWAS catalog

In the study, we aim to compare the prediction performance of six CVD-related diseases under three different scenarios: PRS-only, EHR-only, and PRS + EHR. For the analysis, genetic and clinical data as well as GWAS summary statistics are required.

Genetic and clinical data were obtained from FHS, which was initiated in 1948 in Framingham, Massachusetts, to investigate the long-term development of CVD and identify its underlying causes. This study includes a total of 7,082 participants from four FHS cohorts: 596 from the Original Cohort, 2,754 from the Offspring Cohort, 88 from the New Offspring Spouse Cohort, and 3,644 from the Third Generation Cohort. Clinical examinations were conducted at different follow-up intervals, including 32 for the Original Cohort, nine for the Offspring Cohort, and three each for the New Offspring Spouse and Third Generation Cohorts. Data are available upon approval through dbGaP (phs000007.v32.p13).

GWAS summary statistics for AF, stroke, myocardial ischemia, diastolic heart failure, Alzheimer’s disease, and diabetes were obtained from the GWAS Catalog (https://www.ebi.ac.uk/gwas/): GCST006414 [52], GCST90044350 [53], GCST90473543 [54], GCST90480183 [55], GCST007320 [56], and GCST90267278 [57], respectively. These datasets were used as base data for PRS construction for the corresponding diseases: AF, stroke, CHD, CHF, dementia, and diabetes.

### Preprocessing and quality control

Genotype quality control (QC) was performed using PLINK v1.9. Variants were excluded if they had a call rate < 99%, a minor allele frequency (MAF) < 1%, or deviated from Hardy–Weinberg equilibrium in controls (P < 1 × 10 ⁻ ⁶). Samples with a call rate below 99% were also removed. For PRS analysis, harmonization between base GWAS summary statistics and target genotype data was performed. Variants with allele mismatches, strand inconsistencies, or genomic position discrepancies were excluded, retaining only variants present in both datasets. PRS weights were assigned using complete GWAS summary statistics for each disease outcome. After QC and harmonization, genotype data from 6,170 individuals were retained for dementia, and from 7,082 individuals for all other diseases; these data were then used for downstream analyses. The final number of variants used for PRS construction is shown in Table 1.

**Table 1:** Sample size and number of Variants included in the study after QC.

Trait	Cases	Controls	Number of variants
AF	879	6,203	6,147,094
CHD	889	6,193	6,033,020
CHF	508	6,574	6,153,631
Dementia	420	5,750	6,059,789
Diabetes	804	6,278	6,133,315
Stroke	391	6,691	6,266,023

Trait: Disease analyzed; Cases: Number of participants with the disease; Controls: Number of participants without the disease; Number of variants: Genetic variants retained after QC for analysis.

QC and harmonization procedures were applied to the GWAS summary statistics as well. GCST90473543 and GCST90480183 were originally aligned to genome build GRCh38, whereas the FHS genotype data are based on GRCh37. Therefore, these two datasets were converted to GRCh37 using CrossMap [58] before further analysis. Following the genome build conversion, variant-level QC was conducted by excluding SNPs with MAF < 0.01 or imputation quality score < 0.8. Duplicate SNPs with identical rsIDs were removed. Strand-ambiguous SNPs (A/T or C/G) were excluded to prevent alignment errors. SNP harmonization between the base and target datasets was performed; variants with discordant alleles were removed unless strand flipping resolved the mismatch. Variants with ambiguous alleles relative to the target dataset were further excluded. Table 2 summarizes sample sizes and variant counts after QC.

**Table 2:** GWAS summary statistic information used for the analysis.

Trait	Ancestry	Genome Build	GCST ID	Sample Size (Cases/ Controls)	Number of Variants After QC
AF	European	GRCh37	GCST006414	60,620/ 970,216	6,627,448
Myocardial Ischemia	European	GRCh38	GCST90473543	51,010/ 445,429	7,786,812
Diastolic heart failure	African American or Afro-Caribbean European Hispanic or Latin American	GRCh38	GCST90480183	26,634/589,369	7,716,845
Alzheimer	European	GRCh37	GCST007320	24,087/ 383,378	6,241,300
Diabetes	European	GRCh37	GCST90267278	283,227(continuous)	6,061,417
Stroke	European	GRCh37	GCST90044350	6,986/ 448,317	6,133,316

Trait: Disease reported in the corresponding GWAS study; Ancestry: Population ancestry of GWAS participants; Genome Build: Reference genome version used in the GWAS; GCST ID: GWAS Catalog accession ID; Sample Size (Cases/ Controls): Number of participants included in the GWAS; Number of Variants After QC: Variants retained after QC in this study.

For EHR data, 18 clinical EHR variables were initially considered. Disease onset times were identified using disease-specific Survival and Follow-up Datasets provided by the FHS. For each individual, clinical examination data corresponding to the year of disease diagnosis were used for analysis. When data from the exact diagnosis year were unavailable, data from the closest preceding examination were substituted. Through this process, a comprehensive set of clinical and lifestyle variables was compiled. For each disease-specific dataset, principal component analysis (PCA) was applied independently to reduce dimensionality and mitigate multicollinearity. Principal components explaining at least 90% of the total variance were retained. To further remove redundant information, pairwise correlation analysis was conducted among candidate variables. When the absolute correlation coefficient between two variables exceeded 0.8, the variable with lower explanatory contribution was excluded. Notably, although feature selection was performed independently for each disease outcome, the same set of EHR variables was consistently retained across all outcomes, indicating that these variables robustly satisfied the selection criteria and reflected shared cardiometabolic risk factors underlying multiple disease phenotypes. Consequently, a disease-specific set of EHR features was selected for each outcome, as summarized in Table 3. Age and sex were included as common covariates in all analyses. EHR variables were obtained from the FHS Workthru files, specifically from the Frequently Used Cardiovascular Risk Factors dataset. Alcohol consumption variables were processed and categorized to reflect actual intake levels, using the same calculation approach as described in prior work [59]. After excluding individuals with missing data, the final numbers of cases and controls were as follows: AF (658 cases, 5,220 controls), CHD (461 cases, 5,264 controls), CHF (374 cases, 5,569 controls), dementia (323 cases, 5,213 controls), diabetes (681 cases, 5,386 controls), and stroke (256 cases, 5,681 controls).

**Table 3:** Patient clinical and lifestyle variables from the FHS included in the analysis after PCA values were presented as mean (min, max).

	AF	CHD	CHF	Dementia	Diabetes	Stroke
AGE	57.92 (24, 98)	57.36 (24, 96)	58.30 (24, 98)	58.97 (24, 98)	59.45 (24, 96)	58.31 (24, 98)
ALCOHOL	2.32 (0, 48.45)	2.31 (0, 48.4)	2.28 (0, 48.4)	2.26 (0, 48.4)	2.26 (0, 48.4)	2.28 (0, 48.4)
BG	100.95 (43, 408)	100.68 (43, 408)	101.17 (54, 408)	101.43 (43, 408)	101.75 (53, 408)	101.11 (43, 408)
CALC_LDL	105.15 (15.6, 315)	106.11 (16, 315)	104.34 (16, 315)	103.93 (16, 306)	102.77 (16, 340)	104.34 (16, 306)
CPD	1.53 (0, 80)	1.58 (0, 80)	1.47 (0, 80)	1.31 (0, 60)	1.33 (0, 80)	1.46 (0, 80)
CREAT	0.91 (0.33, 5)	0.91 (0.33, 5.9)	0.92 (0.33, 5.9)	0.92 (0.33, 6.33)	0.92 (0.33, 6.33)	0.92 (0.33, 6.33)
DBP	73.02 (38, 123)	73.32 (38, 123)	72.95 (37, 123)	72.77 (37, 123)	72.63 (37, 123)	72.9 (37, 123)
DLVH	0.01 (0, 1)	0 (0, 1)	0.01 (0, 1)	0.01 (0, 1)	0.01 (0, 1)	0.01 (0, 1)
HDL	58.93 (16, 198)	59.07 (17, 198)	58.93 (16, 198)	58.91 (16, 198)	59.34 (14, 198)	58.89 (16, 198)
HGT	66.12 (54, 78.75)	66.15 (54, 78.75)	66.12 (54, 78.75)	66.08 (54, 78.75)	66.04 (53.25, 78.5)	66.12 (54, 78.75)
HIP	41.27 (30.5, 72.5)	41.28 (30.5, 72.5)	41.27 (30.5, 72.5)	41.27 (30.5, 72.5)	41.28 (30.5, 72.5)	41.26 (30.5, 72.5)
SBP	122.31 (80, 212)	122.08 (80, 215)	122.44 (80, 215)	122.74 (80, 215)	122.65 (80, 215)	122.32 (80, 215)
TRIG	119.76 (29, 5329)	118.47 (29, 5329)	118.84 (29, 5329)	118.71 (27, 5329)	117.92 (29, 5329)	119.15 (29, 5329)
VENT_RT	64.24 (34, 138)	64.39 (34, 138)	64.28 (34, 138)	64.06 (34, 138)	64.28 (34, 138)	64.24 (34, 138)

Abbreviations: BG, blood glucose; CALC_LDL, calculated low-density lipoprotein cholesterol; CPD, cigarettes per day; CREAT, creatinine; DBP, diastolic blood pressure; DLVH, definite left ventricular hypertrophy; HDL, high-density lipoprotein cholesterol; HGT, height; HIP, hip girth; SBP, systolic blood pressure; TRIG, triglycerides; VENT_RT, ventricular beats per minute by electrocardiography.

Among the participants included in the analysis, the number of cases was relatively low compared with controls, resulting in class imbalance. To mitigate this issue during model training, sampling-based imbalance handling strategies were incorporated into the hyperparameter tuning process to identify the most appropriate approach for each model. Details are provided in the “Hyperparameter Tuning for Risk Prediction Models” section.

### PRS

PRS provides a comprehensive estimate of the combined effect of genetic variants on disease occurrence and has been increasingly applied across various diseases, enhancing prediction performance [5,6]. PRS is defined as the sum of risk alleles, each weighted by its corresponding genotype effect size. To calculate the PRS, base and target data sets are required. Base data typically consist of GWAS summary statistics detailing the associations between genetic variants and specific diseases or traits. Target data included genotype and phenotype information from the population under study, which must be independent of the base data. The PRS individual i is calculated using the following equation:


$${PRS}_{i}=\sum_{j=\text{1}}^{M}X_{ij}{\hat{\beta }}_{j}$$

Where ${PRS}_{i}$ represents the score for an individual i, M denotes the number of SNPs, *X* indicates the genotypes of the target data, and $\hat{\beta }$ represents the weights derived from the base data. These weights were determined based on effect size estimates from GWAS and may be adjusted depending on the calculation methods used.

In this study, PRS were calculated using three widely used methods: PRSice2, Lassosum, and LDpred2. These methods represent different modeling strategies for PRS construction and were applied uniformly to the same datasets to enable a fair comparison of predictive performance. For each disease, the PRS derived from the method yielding the highest AUC in logistic regression was selected for analysis. Effect sizes for PRS construction were based on the beta coefficients from the previously described GWAS summary statistics.

### Risk prediction model

Logistic regression and four ML methods were used to develop risk prediction models for CVD based on PRS and EHR data. Logistic regression and four ML methods: random forest, XGBoost, CatBoost, and LightGBM, were applied to identify the best-fitting model for each disease. Random forest is an ensemble learning method that combines multiple decision trees to produce aggregated predictions. It outperforms other ML methods, such as Naïve Bayes and AdaBoost, in previous studies [1,52,53]. XGBoost, CatBoost, and LightGBM are based on the Gradient Boosting algorithm [46–48]. XGBoost improves overall model predictions by iteratively training new models. CatBoost automatically handles categorical features, making it ideal for categorical data. LightGBM uses a leaf-wise tree-splitting strategy to minimize prediction errors. The dataset was split into training (80%) and test (20%) sets for model training and evaluation [54].

### Hyperparameter tuning for risk prediction model

Logistic regression and four ML methods were evaluated for risk prediction. For each model, algorithm-specific hyperparameter search spaces were predefined, and a fully nested cross-validation framework was employed to prevent optimistic bias. The dataset was partitioned using stratified four-fold outer cross-validation, with each outer test fold used exclusively for final performance evaluation.

Within each outer training fold, stratified three-fold inner cross-validation was conducted to jointly optimize model hyperparameters and class imbalance handling strategies, including undersampling, SMOTE-based oversampling, and class-weight adjustment. Sampling ratios of 0.6, 0.8, and 1.0 were explored where applicable. The mean ROC-AUC across inner folds was used as the selection criterion. All resampling procedures and preprocessing steps were performed using training data only to prevent information leakage. Regarding feature preprocessing, age was intentionally retained on its original scale, as it serves as a clinically interpretable risk adjustment covariate rather than a feature whose scale is intended to influence model optimization. Sex and DLVH status were also used in their original form, while all remaining continuous variables were z-score standardized. The algorithm-specific hyperparameter search spaces explored for each model are summarized in S1 Table.

After selecting the optimal configuration, models were retrained on the full outer training set and evaluated on the independent outer test set. Model performance was primarily assessed using ROC-AUC. For clinical interpretability and comparability across models, a fixed classification threshold of 0.5 was applied uniformly to compute sensitivity, specificity, likelihood ratios, and diagnostic odds ratios (DORs). Full results from the nested cross-validation are provided in S1 File.

### Model evaluation metrics

Model performance was evaluated using multiple complementary metrics, including the AUC, area under the precision–recall curve (AUPRC), Brier score, sensitivity, specificity, DOR, positive likelihood ratio (LR+), and negative likelihood ratio (LR−). Discriminative ability was primarily assessed using ROC curves, which plot sensitivity against the false positive rate across varying decision thresholds. The AUC, ranging from 0.5 to 1.0, was used as the main summary measure of discrimination, with higher values indicating improved ability to distinguish individuals with and without disease. To quantify statistical uncertainty, 95% confidence intervals (CIs) for AUC were estimated using DeLong’s method.

Given the presence of class imbalance in several disease outcomes, AUPRC was additionally reported to provide a more informative assessment of predictive performance for the positive class. Unlike AUC, AUPRC emphasizes precision–recall trade-offs and is particularly suitable for imbalanced datasets. Model calibration was evaluated using the Brier score, which measures the mean squared difference between predicted probabilities and observed outcomes, with lower values indicating better calibrated and more accurate probabilistic predictions.

Sensitivity and specificity were calculated to assess the ability of each model to correctly identify diseased and non-diseased individuals, respectively. CIs were estimated to account for sampling variability. Overall diagnostic performance was further summarized using the DOR, derived from sensitivity and specificity, with higher values indicating stronger discriminatory power. In addition, the LR + , defined as sensitivity divided by (1 − specificity), and the LR − , defined as (1 − sensitivity) divided by specificity, were computed. Higher LR+ values and lower LR− values indicate superior diagnostic performance.

### Implementation

All analyses were conducted using R (version 3.6.0) and Python (version 3.10.19). PRS estimation was performed in R using PRSice2, LDpred2, and lassosum; clumping and p-value thresholding were applied only in PRSice2 using default settings, whereas LDpred2 and lassosum leveraged LD information from the HapMap3 reference panel without explicit p-value thresholds. All other analyses, including ML model training and evaluation, were performed in Python. ML methods were implemented using scikit-learn (version 1.7.2), XGBoost (version 2.0.3), LightGBM (version 4.6.0) and CatBoost (version 1.2.8). Class imbalance handling methods, including oversampling and undersampling, were implemented using the imbalanced-learn package (version 0.14.0). Logistic regression models were implemented using the LogisticRegression class from scikit-learn with L2 regularization and the liblinear solver. All random seeds were fixed within each cross-validation procedure to ensure reproducibility, and SHAP (version 0.44.1) values were computed on the held-out test data only to avoid information leakage; for tree-based models, a model-consistent explainer (TreeExplainer) was used.

## Results

### PRS estimation

To select the method for estimating the PRS score, three different PRS computation methods were compared: PRSice2 [60], LDpred2 [61], and lassosum [62]. For each disease outcome, a logistic regression model was fitted using a single PRS variable as the sole predictor. Model performance was assessed using the AUC.

To obtain robust performance estimates, the training and evaluation procedure was repeated 100 times with different random data splits, and the mean AUC across repetitions was used as the final performance metric. Mean AUC values for each PRS method and disease are summarized in Table 4. For each disease, the PRS method achieving the highest mean AUC was selected as the representative genetic risk score and subsequently incorporated into the corresponding prediction model.

**Table 4:** Comparison of AUC (mean, CI) Values Obtained from PRSice2, Ldpred2, and Lassosum.

PRS	AF	Stroke	CHD	CHF	Dementia	Diabetes
PRSice2	0.64 ± 0.03	0.50 ± 0.05	0.44 ± 0.03	0.55 ± 0.03	0.53 ± 0.05	0.42 ± 0.03
LDpred2	0.53 ± 0.03	0.49 ± 0.05	0.52 ± 0.03	0.51 ± 0.05	0.54 ± 0.04	0.59 ± 0.03
Lassosum	0.55 ± 0.03	0.52 ± 0.05	0.50 ± 0.03	0.48 ± 0.03	0.49 ± 0.04	0.56 ± 0.04

Although performance varied across diseases, the method yielding the highest mean AUC was selected for each outcome. PRSice2 was used for AF and CHF; LDpred2 was applied for CHD, dementia and diabetes; and Lassosum was utilized for stroke. Additional model performance metrics, including AUPRC and mean Brier scores, are reported in S2 Table.

### Performance of CVD prediction model

**Fig 2:**
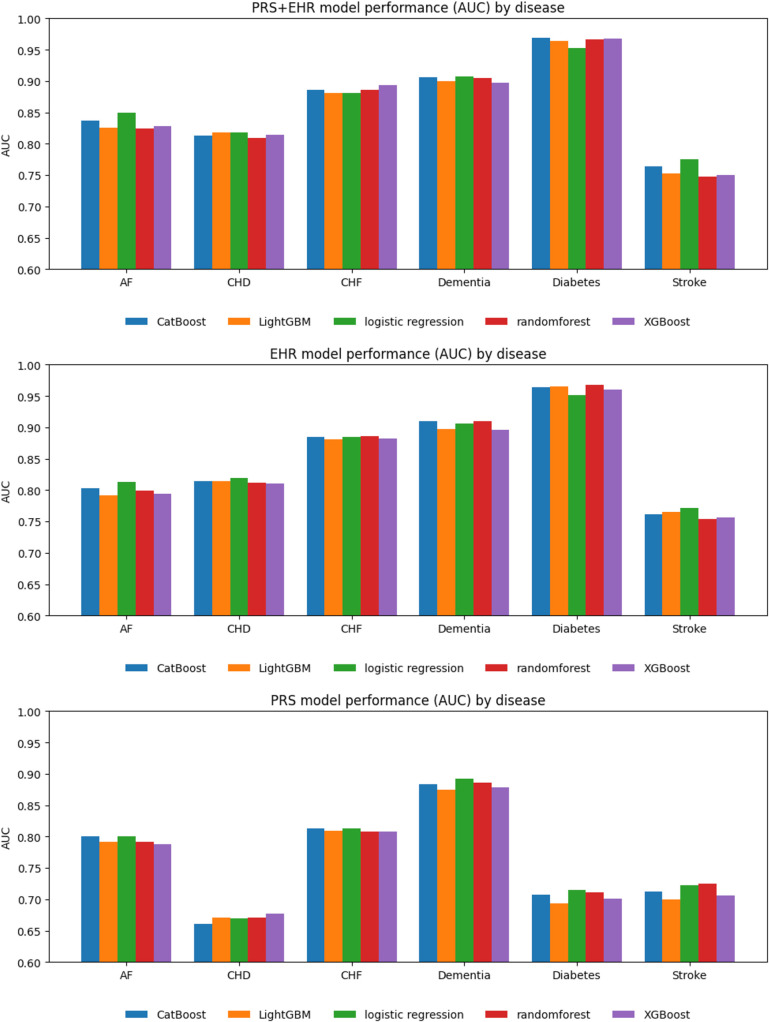
Histograms illustrate AUC values for five prediction methods across six CVD-related diseases under three scenarios. (1) PRS-only, (2) EHR-only and (3) PRS + EHR. Blue, orange, green, red and purple bars represent the AUC values for CatBoost, LightGBM, logistic regression, random forest and XGBoost respectively.

To evaluate the performance of models for predicting six CVD-related conditions: AF, stroke, CHD, CHF, dementia, and diabetes, AUC values were calculated for logistic regression and four ML methods: random forest, XGBoost, CatBoost, and LightGBM. The method achieving the highest AUC value in each case was identified as the best-fit prediction method for the respective disease. The analysis was conducted across three scenarios: (1) PRS-only, (2) EHR-only and (3) PRS + EHR. Five prediction methods were evaluated for each disease under three different scenarios, resulting in 15 combinations of models. Fig 2 presents histograms of AUC values for the five methods across the six diseases under the three scenarios. The full model evaluation metrics are provided in S2 File.

**Fig 3:**
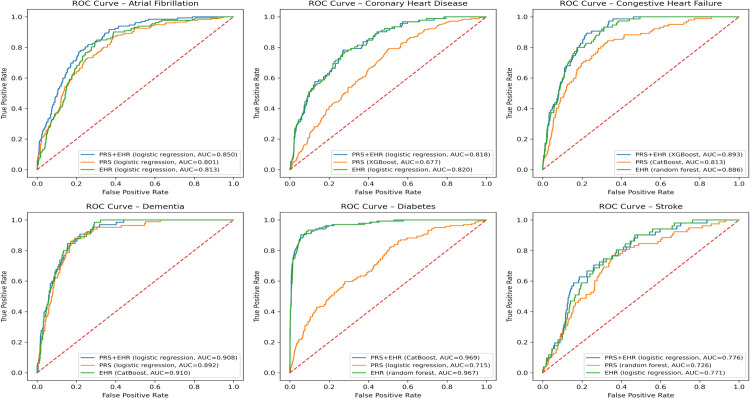
ROC curves for the three scenarios evaluated using the best-fit prediction method for six CVD-related diseases. Blue, orange, and green lines represent (1) PRS-only, (2) EHR-only and (3) PRS + EHR, respectively.

Additionally, the ROC curves for the three scenarios were compared using the best-fit prediction method for each of the six CVD-related diseases (Fig 3).

In addition to AUC, AUPRC, Brier Score, sensitivity, specificity, DOR, LR+ and LR- were compared for the best-fit prediction methods across the three scenarios (Table 5).

**Table 5:** Performance metrics of the best-fit prediction models across three scenarios.

AF	PRS EHR	PRS	EHR	CHD	PRS EHR	PRS	EHR	CHF	PRS EHR	PRS	EHR
AUC	0.850	0.801	0.813	AUC	0.818	0.677	0.820	AUC	0.893	0.812	0.886
AUPRC	0.418	0.372	0.323	AUPRC	0.284	0.210	0.285	AUPRC	0.337	0.258	0.339
Brier Score	0.170	0.193	0.189	Brier Score	0.180	0.223	0.181	Brier Score	0.147	0.172	0.167
Sensitivity	0.803	0.755	0.803	Sensitivity	0.783	0.753	0.772	Sensitivity	0.893	0.765	0.853
Specificity	0.749	0.701	0.720	Specificity	0.728	0.521	0.721	Specificity	0.775	0.725	0.740
DOR	12.17	7.281	10.50	DOR	9.621	3.307	8.740	DOR	28.80	8.556	16.61
LR+	3.200	2.534	2.871	LR+	2.874	1.570	2.767	LR+	3.965	2.778	3.290
LR-	0.263	0.348	0.273	LR-	0.298	0.475	0.317	LR-	0.138	0.325	0.198
Dementia	PRS EHR	PRS	EHR	Diabetes	PRS EHR	PRS	EHR	Stroke	PRS EHR	PRS	EHR
AUC	0.908	0.892	0.910	AUC	0.969	0.715	0.967	AUC	0.776	0.726	0.771
AUPRC	0.328	0.333	0.345	AUPRC	0.852	0.260	0.841	AUPRC	0.132	0.117	0.127
Brier Score	0.129	0.142	0.123	Brier Score	0.055	0.211	0.067	Brier Score	0.203	0.222	0.203
Sensitivity	0.846	0.880	0.877	Sensitivity	0.875	0.627	0.890	Sensitivity	0.706	0.794	0.725
Specificity	0.813	0.791	0.799	Specificity	0.945	0.649	0.931	Specificity	0.695	0.578	0.700
DOR	23.92	28.05	28.26	DOR	120.9	3.121	109.4	DOR	5.464	5.324	6.169
LR+	4.526	4.221	4.355	LR+	15.99	1.790	12.96	LR+	2.313	1.887	2.419
LR-	0.189	0.150	0.154	LR-	0.132	0.573	0.118	LR-	0.423	0.354	0.392

For AF, the PRS + EHR scenario achieved the highest discrimination, outperforming the other scenarios across all evaluation metrics except sensitivity, where it matched the EHR-only scenario. Interestingly, the PRS-only scenario also showed strong performance in AF, underscoring the important role of genetic risk in this condition. Similarly, for CHF, the PRS-only scenario yielded a relatively high AUC of 0.812, and apart from AUPRC, incorporating PRS information appeared to enhance the predictive performance beyond that of EHR alone.

In some diseases, the EHR-only models performed so well that adding PRS provided little additional benefit. For dementia, the PRS-only scenario achieved a fairly high AUC of 0.892, but the EHR-only scenario reached an AUC of 0.910, leaving little room for improvement and resulting in no meaningful gain from combining PRS with EHR. In diabetes, the EHR-only scenario showed extremely strong performance with an AUC of 0.967, whereas the PRS-only scenario had a comparatively lower AUC of 0.715, so adding PRS contributed only modestly to the prediction.

For CHD, the PRS-only scenario showed a relatively modest performance with an AUC of 0.677, while the EHR-only and PRS + EHR scenarios exhibited similar levels of discrimination. For stroke, all three scenarios yielded AUCs in the 0.7 range with no substantial differences, and the best-performing scenario varied by evaluation metric, with PRS + EHR, PRS-only, and EHR-only each showing stronger performance in different aspects.

### Feature importance of genetic and environmental variables in CVD prediction

**Fig 4:**
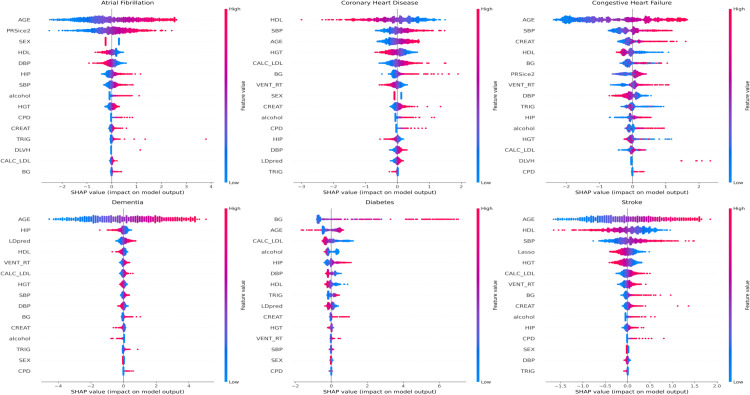
SHAP plot for the PRS + EHR model showing feature contributions to predictions for CVD-related diseases. The figure illustrates the distribution of feature importance and the correlation of predictive variables across six CVDs. The x-axis represents the SHAP values, while the color of the dots indicates the corresponding feature values.

To evaluate feature importance in disease prediction, SHAP analysis was conducted to identify factors that most strongly influenced model outputs and to facilitate interpretability of the prediction results. The analysis was performed for the PRS + EHR models, using the best-performing method selected separately for each disease outcome based on predictive performance. Fig 4 presents the SHAP summary plots highlighting the relative contributions of PRS and clinical features to disease risk prediction across the six diseases.

The analysis revealed that age was the dominant contributor to phenotype prediction, ranking first for four out of the six examined phenotypes while second rank for diabetes and CHD. This phenomenon strongly aligns with the established epidemiological characteristics of CVDs, although it is imperative to note that this predictive dominance does not necessarily imply a direct causal relationship [63–65]. Additionally, a positive contribution of blood glucose (BG) emerged as a dominant predictor for diabetes, clearly presenting a key clinical symptom of diabetes. Concurrently, high-density lipoprotein (HDL) was identified as a protective feature for most CVDs, reflecting its beneficial role in maintaining vascular health [66–68]. Distinct sex-specific contributions were observed in AF where sex exhibited a consistent fixed effect across all epochs [69]. This pattern conforms to clinical data indicating that baseline risk levels for AF and CHF are substantially higher in males than in females.

Beyond clinical parameters, genetic risk features demonstrated varying degrees of contribution depending on the phenotype. For AF, the polygenic risk feature from PRSice2 ranked second, suggesting that genetic predisposition is a critical determinant for this arrhythmia. Similarly, for dementia, the polygenic risk feature from LDpred2 ranked third, showing its increasing risk upon the genetic predisposition. However, genetic predisposition did not strongly drive the prediction of the remaining phenotypes. Consequently, while PRS-only models exhibited moderate performance, the relative contribution of genetic factors diminished significantly upon the inclusion of robust clinical predictors in the integrated models [64,70].

## Discussion

CVD remains a leading cause of mortality worldwide and contributes substantially to escalating healthcare expenditures. Effective management of modifiable risk factors can markedly reduce their burden in many cases. Over the past decade, risk prediction methodologies have evolved considerably, particularly with the introduction of PRS, which are often combined with clinical information to enhance prediction. Nonetheless, most existing studies have focused on single endpoints, such as specific cardiac events. Given that CVD arises from a complex interplay of genetic predisposition and environmental influences, a broader, more integrated evaluation of prediction performance is needed. Using data from the FHS, we examined how genetic and clinical factors jointly contribute to cardiovascular risk. We compared multiple prediction models across different scenarios for six cardiovascular-related diseases and evaluated their performance using various metrics. We also assessed the importance of individual genetic and clinical variables and found that the relative contributions varied by disease. Overall, our findings indicate that the relative contributions of PRS and EHR variables differ substantially by disease, and that the method of integrating these data sources can influence both predictive performance and interpretation.

Across many CVD-related outcomes, the EHR-only scenario achieved high discrimination, leaving limited room for improvement by adding PRS. For dementia, the PRS-only scenario yielded a strong AUC of 0.892; however, the EHR-only scenario performed better (AUC = 0.910), and combining PRS with EHR did not produce additional gains. For diabetes, the EHR-only scenario reached an AUC of 0.967, whereas the PRS-only scenario achieved an AUC of 0.715. The incremental benefit of adding PRS was modest, likely reflecting the presence of highly informative clinical variables such as BG levels in the EHR. In contrast, PRS played a more prominent role in CHF and AF, where adding PRS to EHR data improved predictive performance, suggesting that integrating genetic information with clinical data can provide additional value. Notably, PRS alone achieved an AUC greater than 0.8 for AF, CHF, and dementia, suggesting that genetic information may have substantial diagnostic utility for these conditions. This pattern was also reflected in the SHAP analysis, where genetic risk features ranked among the top predictors for these diseases. In AF, genetic factors accounted for a large proportion of feature importance, consistent with prior estimates of clinical heritability (derived from twin or family studies) and genetic heritability (*h*^*2*^_*g*_) [71,72]. For example, a 2009 Danish twin study using a biometric model with environmental and additive genetic components estimated the heritability of AF at 62% [73]. Similarly, elevated genetic risk for dementia has been reported in large-scale population genetic studies [74–76]. By contrast, the PRS-only scenario demonstrated relatively modest performance for CHD. In this context, the EHR-only and PRS + EHR scenarios exhibited similar discrimination, suggesting that clinical and environmental factors captured in the EHR may be more influential for risk prediction. There were also cases in which no single scenario consistently dominated across metrics. For stroke, all three scenarios produced AUCs in the 0.7 range, and the best-performing scenario varied by metric. This finding suggests that, with the current data, no single information source clearly outperforms the others.

Collectively, these results highlight the need for disease-specific strategies when integrating genetic and clinical information. Integrating PRS with EHR data generally improves predictive performance compared with using either PRS or EHR alone. However, for diseases in which EHR-based prediction is already highly accurate, the added value of PRS may be limited. Conversely, for conditions such as AF and CHF, PRS can provide substantial predictive information, either alone or in combination with EHR. These insights can inform the design of future risk prediction models by helping to prioritize when PRS should be incorporated and when investment in richer clinical data may yield greater gains.

Some limitations of this study should be acknowledged. First, the FHS predominantly comprises individuals of European ancestry, which may limit the generalizability of our findings to populations with different genetic backgrounds and environmental exposures. External validation in independent, multi-ethnic cohorts will be essential to assess the robustness and transferability of the proposed models. Second, although we applied a nested cross-validation framework and performed all preprocessing steps within the training folds to minimize information leakage, residual sources of leakage cannot be completely excluded. In particular, explicit family or pedigree information was not available in the dataset, preventing family-aware data partitioning. As a result, related individuals may have been included across training and test folds, potentially leading to optimistic performance estimates due to shared genetic and environmental factors. Future studies incorporating family identifiers should evaluate model performance using family-based or cluster-level validation strategies. Third, although SHAP analysis was employed to enhance interpretability and identify features that strongly influenced model predictions, these importance scores should not be interpreted as evidence of causal relationships. SHAP values reflect model-dependent associations rather than underlying biological causation; therefore, conclusions regarding causality should be drawn with caution. In addition, formal uncertainty estimation for SHAP values (e.g., via bootstrapping) was not performed, which limits the robustness and quantitative interpretability of the reported feature importance. Additional limitations include the lack of benchmarking against established clinical risk equations, such as the Framingham risk scores, and the absence of formal calibration and clinical utility analyses, including calibration-in-the-large, net reclassification improvement, integrated discrimination improvement, and decision curve analysis. Furthermore, the feature set was restricted to variables available in the FHS Workthru datasets, and the analyses relied on a single cohort, which may limit the breadth of clinical and environmental factors captured. Addressing these limitations through external validation, expanded feature sets, and comprehensive clinical utility assessments will be important directions for future research.

## Conclusion

This study systematically evaluated the predictive performance of PRS, EHR–based clinical variables, and their integration across six CVD–related outcomes using data from the FHS. By comparing multiple prediction models under PRS-only, EHR-only, and PRS + EHR scenarios, we demonstrated that the relative contribution of genetic and clinical information to disease risk prediction varies substantially across diseases.

Overall, EHR-based models achieved strong discrimination for several outcomes, particularly diabetes and dementia, leaving limited room for improvement through the addition of PRS. In these settings, highly informative clinical variables derived from routine care dominated predictive performance, and the incremental value of genetic risk information was modest. In contrast, integrating PRS with EHR data improved prediction accuracyfor AF and CHF, highlighting the complementary role of genetic information when clinical predictors alone are insufficient. Notably, PRS-only models achieved substantial discrimination for certain highly heritable conditions, suggesting that genetic information may provide meaningful predictive utility even in the absence of detailed clinical data.

Feature importance analysis using SHAP further indicated that age and key clinical variables were the dominant predictors across most disease outcomes, while the contribution of genetic risk varied by phenotype. These patterns are consistent with established epidemiological and heritability evidence and emphasize that predictive importance does not necessarily imply causality. Notably, the reduced relative contribution of PRS in the presence of strong clinical predictors underscores that the value of genetic information depends on both disease etiology and the availability and quality of EHR data.

In summary, the findings indicate that a uniform strategy for integrating PRS into cardiovascular risk prediction is unlikely to be optimal. Instead, disease-specific modeling approaches are warranted, with PRS offering the greatest benefit for conditions with substantial genetic susceptibility or limited clinical predictors. These insights provide practical guidance for developing future risk prediction models and for determining when incorporating genetic information is most likely to yield clinically meaningful improvements.

## Supporting information

S1 Table Hyperparameter search spaces for each model. The hyperparameters and value ranges evaluated during model development are listed for each algorithm.
(DOCX)

S2 Table AUC, AUPRC, and Brier mean scores for PRSice2, LDpred2, and Lassosum across diseases. The table reports the mean AUC, standard deviation, percentile-based confidence intervals, AUPRC, and Brier score for each model and disease.
(DOCX)

S1 File Full results from the nested cross-validation. (ZIP)

S2 File Additional model performance metrics from PRSice2, Ldpred2, and Lassosum. (ZIP)

## Abbreviations

AF: atrial fibrillation
AUC: area under the receiver operating characteristic curve
AUPRC: area under the precision–recall curve
BG: blood glucose
CALC_LDL: calculated low-density lipoprotein cholesterol
CHD: coronary heart disease
CHF: congestive heart failure
CI: confidence interval
CPD: cigarettes per day
CREAT: creatinine
CVD: cardiovascular disease
DBP: diastolic blood pressure
DLVH: definite left ventricular hypertrophy
DOR: diagnostic odds ratio
EHR: electronic health records
FHS: Framingham Heart Study
GWAS: genome-wide association studies
HDL: high-density lipoprotein cholesterol
HGT: height
HIP: hip girth
LR+: positive likelihood ratio
LR−: negative likelihood ratio
MAF: minor allele frequency
ML: machine learning
PCA: principal component analysis
PRS: polygenic risk score
QC: quality control
SBP: systolic blood pressure
SHAP: Shapley Additive Explanations
TRIG: triglycerides
VENT_RT: ventricular rate by electrocardiography
