# Supplementary material for: Risk prediction for cardiovascular related diseases using PRS and EHR in the Framingham Heart Study
Source: PLoS One. 2026 Apr 17;21(4):e0345914. doi: 10.1371/journal.pone.0345914 (PMC13089760; doi:10.1371/journal.pone.0345914)
Supplement: S1 Table — The hyperparameters and value ranges evaluated during model development are listed for each algorithm. (DOCX) [file pone.0345914.s001.docx]

**S1 Table.** **Hyperparameter search spaces for each model**

| **Model** | **Hyperparameter** | **Values explored** |
| --- | --- | --- |
| Logistic Regression | C | 0.01, 0.1, 1.0, 10 |
|  | Penalty | L2 |
|  | Solver | liblinear |
| Random Forest | n_estimators | 100, 300, 500 |
|  | max_depth | 3, 5, 10 |
|  | min_samples_split | 2, 5, 10 |
|  | min_samples_leaf | 1, 2 |
|  | max_features | sqrt, log2 |
|  | criterion | gini, entropy |
| XGBoost | n_estimators | 100, 300, 500 |
|  | max_depth | 3, 5, 10 |
|  | learning_rate | 0.1, 0.3 |
| LightGBM | subsample | 0.6, 1.0 |
|  | colsample_bytree | 0.7, 1.0 |
|  | n_estimators | 100, 300, 500 |
|  | max_depth | 3, 5, 10 |
|  | num_leaves | 31, 63 |
|  | learning_rate | 0.1, 0.3 |
|  | subsample | 0.6, 1.0 |
|  | colsample_bytree | 0.7, 1.0 |
| CatBoost | iterations | 100, 300, 500 |
|  | depth | 5, 10 |
|  | learning_rate | 0.1, 0.3 |
|  | l2_leaf_reg | 1, 3 |

The hyperparameters and value ranges evaluated during model development are listed for each algorithm.
