# Supplementary material for: Risk prediction for cardiovascular related diseases using PRS and EHR in the Framingham Heart Study
Source: PLoS One. 2026 Apr 17;21(4):e0345914. doi: 10.1371/journal.pone.0345914 (PMC13089760; doi:10.1371/journal.pone.0345914)
Supplement: S2 Table — The table reports the mean AUC, standard deviation, percentile-based confidence intervals, AUPRC, and Brier score for each model and disease. (DOCX) [file pone.0345914.s002.docx]

**S2 Table. AUC, AUPRC, and Brier mean scores for PRSice2, LDpred2, and Lassosum across diseases**

| **disease** | **model** | **AUC_mean** | **AUC_sd** | **AUC_p025** | **AUC_p975** | **AUPRC_mean** | **Brier_mean** |
| --- | --- | --- | --- | --- | --- | --- | --- |
| AF | PRSice2 | 0.644409 | 0.017022 | 0.610738 | 0.677714 | 0.246786 | 0.108801 |
| AF | Ldpred2 | 0.525203 | 0.016512 | 0.496426 | 0.553319 | 0.139253 | 0.10876 |
| AF | Lassosum | 0.545061 | 0.016182 | 0.512463 | 0.576795 | 0.149675 | 0.108504 |
| CHD | PRSice2 | 0.441876 | 0.016326 | 0.409016 | 0.473435 | 0.10993 | 0.10986 |
| CHD | LDpred | 0.520017 | 0.01461 | 0.493355 | 0.548357 | 0.139299 | 0.109842 |
| CHD | Lasso | 0.495556 | 0.015123 | 0.46693 | 0.517153 | 0.128567 | 0.10989 |
| CHF | PRSice2 | 0.554057 | 0.019121 | 0.522844 | 0.592509 | 0.088512 | 0.066413 |
| CHF | LDpred | 0.516662 | 0.024514 | 0.466258 | 0.556974 | 0.078737 | 0.066421 |
| CHF | Lasso | 0.484005 | 0.015476 | 0.449506 | 0.506521 | 0.072842 | 0.066439 |
| Dementia | PRSice2 | 0.53397 | 0.023837 | 0.487325 | 0.579946 | 0.086949 | 0.063438 |
| Dementia | Ldpred2 | 0.535542 | 0.021799 | 0.494787 | 0.575919 | 0.082783 | 0.063405 |
| Dementia | Lassosum | 0.487575 | 0.024873 | 0.439106 | 0.524596 | 0.070202 | 0.063457 |
| Diabet | PRSice2 | 0.421705 | 0.015729 | 0.391513 | 0.452748 | 0.096386 | 0.10055 |
| Diabet | Ldpred2 | 0.589941 | 0.018313 | 0.560253 | 0.62627 | 0.162136 | 0.100094 |
| Diabet | Lassosum | 0.555911 | 0.016973 | 0.519434 | 0.591382 | 0.143586 | 0.100181 |
| Stroke | PRSice2 | 0.495194 | 0.027927 | 0.440924 | 0.543684 | 0.062152 | 0.052027 |
| Stroke | Ldpred2 | 0.485364 | 0.034614 | 0.42185 | 0.531032 | 0.057268 | 0.052027 |
| Stroke | Lassosum | 0.522737 | 0.023875 | 0.473825 | 0.56414 | 0.067865 | 0.052021 |

The table reports the mean AUC, standard deviation, percentile-based confidence intervals, AUPRC, and Brier score for each model and disease.
